# Gender, hormone therapy, and HIV: what should cardiologists know?

**DOI:** 10.1007/s12471-019-1233-6

**Published:** 2019-01-22

**Authors:** C. A. Martinez, R. R. Rikhi

**Affiliations:** Department of Medicine, Miller School of Medicine, University of Miami, Miami, FL USA

**Keywords:** Cardiovascular disease, Hormone replacement therapy, Transgender persons, HIV

## Abstract

Transgender individuals represent the fastest growing minority in the United States and are disproportionately affected by HIV. Hormone therapy is the most common treatment for gender dysphoria in transgender individuals. As HIV is an independent risk factor for coronary artery disease, it is critical to further research the influence masculinising and feminising hormone therapies have on cardiovascular disease. There is a clinical need for evidence-based guidelines for cardiologists to follow to effectively care for and treat transgender patients. For this to be done, the interplay between HIV, hormone therapy, and cardiovascular disease must be better understood through collaboration between researchers and clinicians to achieve maximum benefit from recent advancements.

## Introduction

Biological sex has been recognised as an independent risk factor for the development of cardiovascular disease (CVD) [1]. In recent years, there has been an increased awareness regarding the spectrum of gender diversity and the role of gender as a CVD risk factor, independent of biological sex [1]. Transgender is an overarching term to describe individuals whose gender identity is different from their sex assigned at birth [1]. They play an integral role within the spectrum of gender diversity and are becoming the fastest growing minority in the United States, with more than 1.4 million individuals identifying as transgender [2]. There is a growing body of research investigating health disparities in this population; yet, limited information exists regarding the cardiovascular risk profiles of transgender men and women. Thus, current cardiac risk stratification tools are complicated and inaccurate because the confounding effect of exogenous hormone therapy on biological sex-based cardiac risk calculators is unknown [1]. This is highly concerning for the current medical society, since there are limited evidence-based recommendations to guide management for CVD prevention in the transgender community.

There are two relevant aspects that should be recognised when providing care for transgender individuals: (1) more than 75% of transgender men and women are on either masculinising or feminising hormone therapy, respectively, and (2) they are disproportionately affected by the HIV epidemic [1]. Although HIV is an important risk factor for the development of CVD, there is a controversial understanding of the cardiovascular effects of concomitant hormone therapy.

## Hormones and CVD at a cellular level

Seminal research has focused on understanding the cellular interaction of masculinising and feminising hormones on the vasculature. Endogenous hormones directly influence vascular endothelial function through androgen receptors (ARs) and oestrogen receptors (ERs) present on endothelial cells [3]. After ligand binding, oestrogen and testosterone translocate into the nuclei of endothelial cells, leading to genomic effects [4]. Under physiological conditions, endogenous activation of both ARs and ERs leads to upregulation of athero-protective genes and downregulation of pro-atherogenic genes [4]. Additionally, activation of both receptors has been shown to have non-genomic effects, by directly upregulating endothelial nitric oxide synthesis, which mediates vasodilatory function [3]. However, these effects have not been translated into clinical outcomes.

## Hormones and CVD in cisgender individuals

Despite the expansive use of hormone therapy in cisgender individuals, conflicting evidence still exists regarding their cardiovascular effects [4]. Premenopausal women have a lower incidence of coronary artery disease than age-matched cisgender men and postmenopausal women, suggesting the cardioprotective effects of endogenous oestrogen [4]. However, the effects of oestrogen replacement therapy continue to be controversial. The Women’s Health Initiative Hormone Therapy Trials found an 18% increase in coronary heart disease in the oestrogen plus progestin group and no difference in the oestrogen only group, when compared to placebo [5]. In contrast, longer follow-up of the younger participants in this trial revealed that oestrogen therapy improved atherosclerosis outcomes [5]. Data on the effect of hormone therapy in cisgender men also suggest an elevated CVD risk. Although testosterone replacement therapy has been used to treat hypogonadism in men, animal and human studies have shown that testosterone treatment for more than 12 weeks increases cardiovascular morbidity and mortality [6]. Testosterone use for more than 1 year in cisgender men has also been associated with increased coronary artery plaque volume when compared to placebo [7]. Nevertheless, data from these trials are not applicable to transgender individuals receiving feminising or masculinising hormone therapy because the clinical characteristics of the study population and hormone regimens differ vastly [1].

## Hormones and CVD in transgender individuals

Many transgender individuals seek long-term hormone therapy to promote physical characteristics that align with their gender identity and to suppress effects of endogenous hormones that are discordant to their identified gender [6]. To date, very few studies have examined the cardiovascular effects of masculinising and feminising hormones in transgender individuals; and those that exist are mired in contradictory results. Masculinising and feminising hormones have been reported to both impair and improve several cardiovascular risk markers [6]. A few studies examining cardiovascular risk among transgender individuals have indicated an increase in hypertension, dyslipidaemia, and diabetes with long-term hormone therapy [6, 8]. A retrospective study in transgender women suggested an increase in cardiovascular morbidity and mortality; however, this has not been demonstrated for transgender men [1, 6, 8]. In addition, these studies are mainly retrospective with several limitations, such as not controlling for smoking or other CVD variables, as well as lacking transgender control groups [1]. In view of the limited available evidence, current guidelines for the primary and gender-affirming care of transgender and gender non-binary people state that CVD risk is unchanged in transgender men, and that the evidence is less clear for transgender women [1].

## HIV and CVD

HIV is now recognised as a CVD risk factor, as it causes immune dysfunction and a heightened inflammatory state that accelerates the development of CVD pathogenesis [9, 10]. Transgender individuals are the highest at-risk population for HIV infection [1]. With the advent of antiretroviral medication therapy, all HIV-infected individuals are living longer and, consequently, have an increased risk for cardiovascular complications [11]. However, HIV-infected persons have elevated subclinical cardiometabolic complications occurring 10–15 years in advance compared to seronegative counterparts [9, 10]. These subclinical cardiometabolic complications include central adiposity, dyslipidaemia, glucose intolerance, and hypertension [9]. There is conflicting evidence regarding primary and secondary prevention guidelines for HIV-infected individuals [12]. In part, this is due to limited information regarding interactions between cardiovascular preventative medications (i. e. antiplatelet and lipid-lowering medications) and antiretroviral therapies [12]. Further, studies have shown that dyslipidaemia may independently interact with HIV to increase inflammation [12]. Biological sex has been shown to impact the effect of HIV on CVD, as studies have shown biological females with HIV to have twice the risk of CVD compared to biological males with HIV [10]. Of great concern is that transgender men and women living with HIV have higher rates of overall morbidity and earlier mortality compared to cisgender HIV-infected individuals [2]. It is not known if CVD risk contributes to this health disparity.

## HIV, hormones, and CVD

**Fig. 1:**
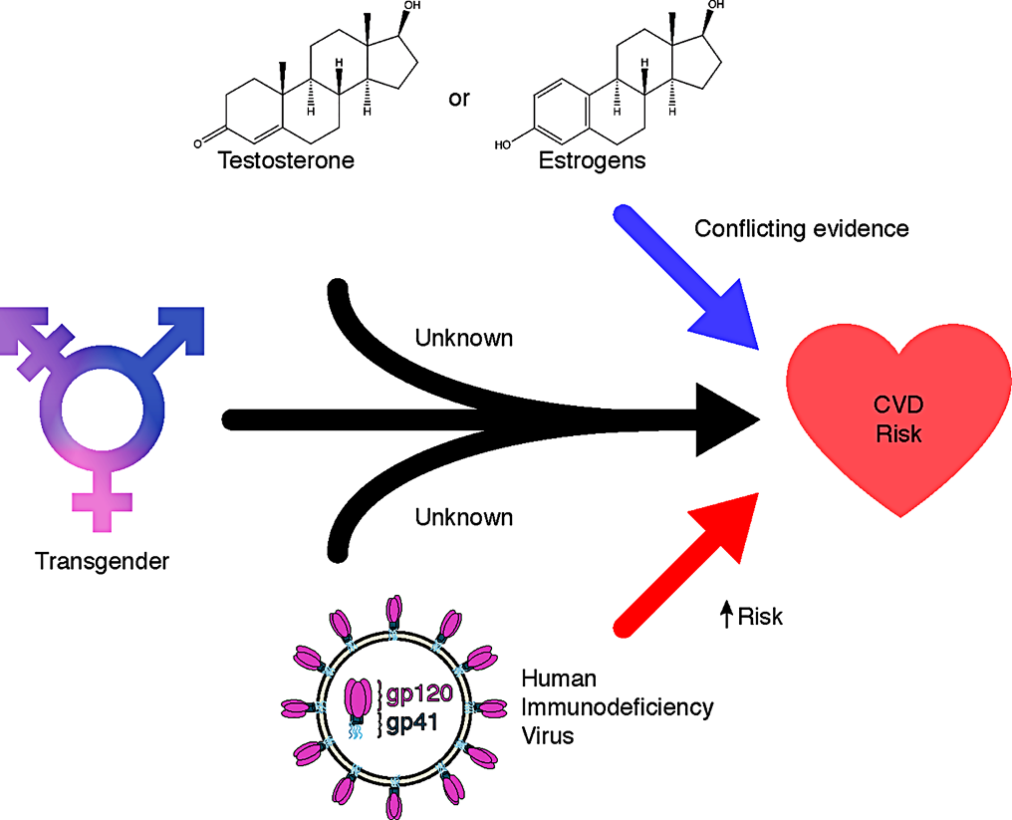
Transgender individuals are disproportionately affected by HIV and an increasing number are exposed to hormone therapy. The cardiovascular effects of HIV and hormone therapy among transgender individuals are not known

There is a paucity of evidence indicating whether HIV-related CVD risk differs in transgender individuals prescribed hormone therapy (Fig. 1). Therefore, a greater awareness of the potential to modify cardiometabolic risk factors associated with both HIV and hormone therapy is encouraged, as it will help decrease the potential CVD burden in this population [9].

## Current treatment

While there are ongoing studies on primary prevention in patients with HIV [13, 14], there are no studies focussing specifically on cardiovascular prevention among transgender persons with HIV. However, there are certain strategies that can be implemented and are applicable to this population, such as encouraging smoking cessation, lipid monitoring and appropriate treatment with a statin that has limited drug interactions with antiretroviral therapy, appropriate selection of antiplatelet therapy, maintaining blood pressure and glucose control, and regular exercise and optimal body mass index. In addition, hormone therapy regimens with the least cardiovascular adverse effects should be used and hormone levels should be monitored to avoid supra-physiological levels that may be associated with a higher CVD risk [15].

## Conclusion

A large gap exists in our understanding of how to effectively provide evidence-based care for transgender individuals. This is a vulnerable population that not only faces constantly shifting social policies, but also clinical challenges due to the complex interplay of CVD risk factors, HIV, and hormone therapy. A multi-disciplinary team approach must focus on addressing unique health care needs among the transgender community. Clinicians together with researchers must join to help narrow this gap and obtain answers that will help develop public health interventions specifically for this medically underserved population.
